# From cats to cortex: *T. gondii* and psychosis, depression, and anxiety

**DOI:** 10.3389/fpsyt.2026.1764597

**Published:** 2026-05-13

**Authors:** Gabriel Andrade, Abderrahim Benlahcene, Dalia Bedewy

**Affiliations:** 1 Department of Basic Sciences, College of Medicine, Ajman University, Ajman, United Arab Emirates; 2 Department of Psychology, College of Humanities and Sciences, Ajman University, Ajman, United Arab Emirates; 3 Humanities and Social Sciences Research Center (HSSRC), Ajman University, Ajman, United Arab Emirates; 4 Department of Educational Psychology, Faculty of Education, Tanta University, Tanta, Egypt

**Keywords:** anxiety, cat ownership, depression, psychiatric disorders, schizophrenia, *T. gondii*

## Abstract

This review examines whether cat ownership, via exposure to the neurotropic parasite *T. gondii*, contributes to vulnerability for psychotic, depressive, and anxiety symptoms. *T. gondii* establishes lifelong latent infection in the brain and muscle, where it can modulate dopaminergic signaling, neuroinflammation, and tryptophan–kynurenine metabolism, providing a biologically plausible pathway to altered cognition, mood, and behavior. Epidemiological and meta-analytic data indicate small-to-moderate associations between *T. gondii* seropositivity and schizophrenia, with more variable but suggestive links to depression and anxiety. Evidence for cat ownership as an independent risk factor is inconsistent: some cohorts and recent meta-analyses report elevated odds of schizophrenia-related outcomes in those exposed to cats, whereas rigorously controlled studies frequently find attenuated or null effects. Methodological limitations, alternative explanations, and cultural implications are discussed, and priorities for mechanism-informed, longitudinal and interventional research are outlined.

## Introduction

In recent years, the intersection of infectious diseases and mental health has become a growing field bridging psychiatry, immunology, and microbiology. Evidence indicates that microorganisms may subtly affect cognition, mood, and behavior through neurobiological pathways (1). Among them, Toxoplasma gondii—a neurotropic protozoan parasite transmitted mainly through cats—has drawn particular attention for its possible role in psychotic and affective disorders. Cat ownership, while emotionally meaningful, might also represent an underrecognized route of exposure to a pathogen with neuropsychiatric implications. Examining this link offers insight into how biological and environmental factors intersect in shaping mental health outcomes.

T. gondii is an obligate intracellular parasite with a life cycle involving felines as definitive hosts and nearly all warm-blooded animals as intermediate hosts (2). Humans acquire infection mainly through ingesting oocysts from cat feces, undercooked meat, or contaminated soil and water. Seroprevalence ranges from 10% to over 60% globally, influenced by geography and dietary habits (3). In addition to the three classical lineages (types I–III), atypical strains have been increasingly recognized and linked to severe disease, including ocular and central nervous system forms (3). Strain diversity may therefore be relevant when evaluating T. gondii–related mental health risks.

Following acute infection, T. gondii establishes a latent form in neural tissue, persisting as bradyzoite cysts capable of altering neurotransmitter systems and neuroinflammatory processes (4). Its affinity for brain regions involved in emotion and cognition—such as the amygdala, hippocampus, and prefrontal cortex—supports hypotheses linking infection to behavioral or psychiatric changes (5). Empirical studies have repeatedly shown higher rates of T. gondii antibodies in individuals with schizophrenia compared to controls (6, 7). Proposed mechanisms include neuroinflammation, immune dysregulation, and dopaminergic disruption, as T. gondii encodes enzymes that may elevate dopamine synthesis (8). Chronic infection may also influence mood through immune activation and altered tryptophan–kynurenine metabolism (9).

**Figure 1:**
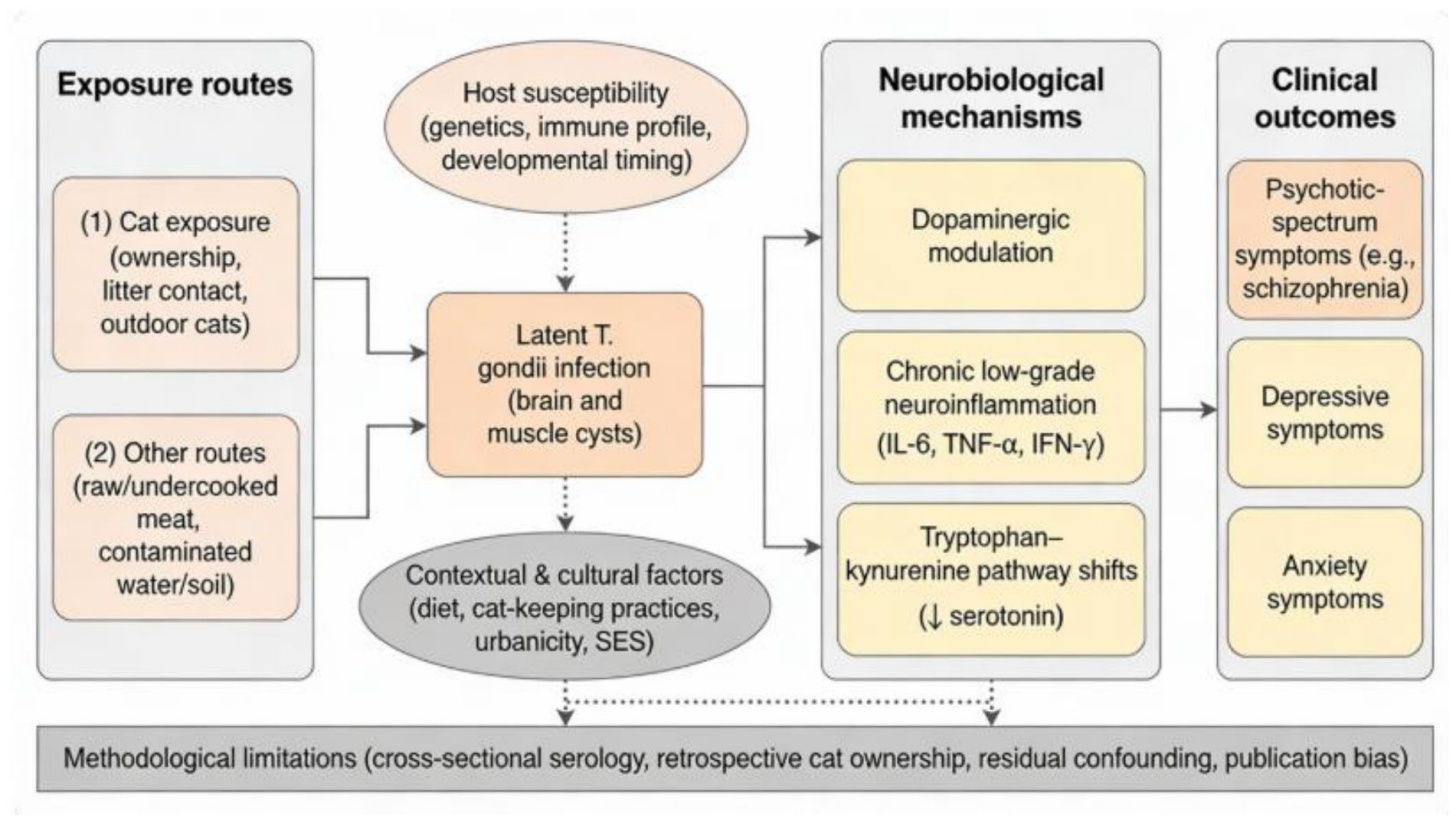
Pathways from *T. gondii* to psychopathology.

Given increasing pet ownership and mental health concerns, the potential mediating role of T. gondii infection in the relationship between cat exposure and psychiatric vulnerability warrants renewed investigation. This review synthesizes epidemiological, clinical, and mechanistic evidence to address three key questions: (1) How strong and consistent is evidence linking T. gondii with psychotic, depressive, and anxiety symptoms? (2) Under what circumstances can cat ownership serve as a meaningful proxy for exposure? (3) Which neurobiological mechanisms mediate these associations and inform broader models connecting infection with mental health? These relationships are summarized in **Figure 1**.

## Methodological transparency statement

This mini-review was conducted as a narrative synthesis of peer-reviewed literature on Toxoplasma gondii, cat ownership, and psychiatric outcomes, prioritizing human studies while incorporating key mechanistic evidence from animal models. Articles were identified through targeted searches of major biomedical databases (e.g., PubMed, PsycINFO) and backward citation tracking, with emphasis on recent, methodologically robust studies, although no formal systematic review protocol or quantitative meta-analysis was undertaken.

## Discussion

### Evidence for Toxoplasma-psychosis association

Epidemiological evidence linking Toxoplasma gondii infection with psychotic disorders, particularly schizophrenia, has accumulated over several decades. Early serological surveys found higher rates of T. gondii antibodies among individuals with schizophrenia than among healthy controls, suggesting an infectious contribution to a multifactorial etiology (10). Large case–control and population-based studies in diverse settings have broadly replicated this pattern; for example, a Danish birth cohort reported that T. gondii seropositivity was associated with roughly a twofold increase in schizophrenia risk after controlling for socioeconomic and perinatal factors (11). Meta-analytic work provides convergent quantitative support: Sutterland et al. (12) reported a pooled odds ratio of 1.81 for schizophrenia among T. gondii–seropositive individuals, and elevated seroprevalence has also been described in bipolar and other psychotic-spectrum conditions, with higher antibody titers sometimes correlating with greater symptom severity and more frequent psychotic episodes (12, 13). Temporal data strengthen causal inference, as prospective cohorts indicate that T. gondii exposure typically precedes illness onset by several years, maternal infection in pregnancy has been linked to increased schizophrenia risk in offspring, and parasite-related immune activation during adolescence—a key period for brain maturation—may heighten vulnerability to later psychosis (14, 15). At the same time, heterogeneity in diagnostic criteria, serological assays, and confounder adjustment has produced null findings in some large datasets, particularly when urbanicity and socioeconomic status are rigorously controlled (16). Overall, the balance of evidence supports an association between latent toxoplasmosis and psychotic phenomena, but one moderated by host susceptibility and environmental context rather than a single, deterministic causal pathway.

### Cat ownership as a risk factor

While T. gondii infection offers a biologically plausible mechanism for infection-related psychosis, the epidemiological evidence implicating cat ownership itself as a risk factor is more inconsistent. Several studies report modest but significant associations between early-life cat ownership and later schizophrenia-spectrum disorders; for example, analyses of two U.S. cohorts found higher odds of schizophrenia among individuals who owned cats in childhood, particularly when exposure occurred before adolescence, a period of heightened neurodevelopmental vulnerability (17, 18). Other investigations, however, including work using U.K. birth cohort data, have found no significant association between childhood cat ownership and psychotic experiences at age 18 once sociodemographic and familial variables are controlled (19). Methodological limitations likely contribute to these discrepancies: most studies use retrospective self-reports of pet ownership rather than direct serological confirmation of T. gondii exposure, and typically do not quantify duration or intensity of cat contact (for example, indoor vs. outdoor cats, litter box cleaning frequency), all of which complicates causal interpretation. Contextual factors such as socioeconomic status, urbanicity, pet hygiene, dietary patterns, and exposure to other animals or contaminated environments shape both T. gondii risk and psychiatric outcomes, and studies reporting positive associations often adjust incompletely for these confounders, whereas larger and more rigorous cohorts tend to yield attenuated or null effects (20, 21).

Crucially, cats represent only one among multiple routes of T. gondii transmission, limiting the specificity of cat ownership as a proxy for infection. Humans can acquire toxoplasmosis not only through ingestion of sporulated oocysts from contaminated soil, food, or water, but also via consumption of raw or undercooked meat containing viable tissue cysts—a major or even predominant source of infection in many culinary contexts favoring undercooked lamb, pork, or game (22, 23). Additional pathways, including unpasteurized milk, inadequately washed vegetables, and occupational exposure in farming or slaughterhouse environments, further underscore that toxoplasmosis is a broadly foodborne and environmental zoonosis rather than a uniquely cat-borne disease (24). Because T. gondii cycles through predator–prey interactions, human carnivorous practices—especially where meat inspection is suboptimal or cultural norms encourage tasting raw minced meat or consuming traditional raw meat dishes—play a central role in sustaining transmission, meaning that cat ownership cannot be assumed to represent the dominant infection route in all populations (25). Recognizing these multiple transmission routes is essential for interpreting associations between T. gondii seropositivity, cat ownership, and psychiatric outcomes, and highlights the need for prospective studies that integrate detailed assessments of dietary habits, food safety behaviors, occupational exposures, and pet-related variables alongside serological measures to disentangle the relative contributions of carnivorous and felid-mediated pathways to both infection risk and putative neuropsychiatric sequelae.

### Beyond serology: molecular insights into *T. gondii*

Beyond serology, a smaller but informative literature has examined molecular detection of Toxoplasma gondii in psychiatric populations, with mixed and sometimes contradictory results. Omar et al. (26) combined serological and PCR-based approaches in schizophrenia patients and showed that, even among seropositive individuals, circulating parasite DNA in peripheral blood was rarely detectable, suggesting that latent infection seldom manifests as measurable parasitemia in these cohorts. Del Grande et al. (27) similarly noted that, although several studies report elevated antibody prevalence in bipolar disorder, attempts to detect T. gondii DNA in blood or cerebrospinal fluid often produce negative or inconsistent findings, a pattern consistent with predominantly latent, neurotropic infection whose peripheral burden lies below standard PCR detection thresholds. In line with this, Galli et al. (28) reported high seroprevalence but no detectable circulating parasite DNA in Italian psychiatric inpatients with schizophrenia or bipolar spectrum disorders, arguing that molecular assays capture only a narrow temporal window of active replication or dissemination and therefore cannot, on their own, adjudicate causal claims. Collectively, these findings indicate that serology alone cannot fully characterize the T. gondii–psychiatry relationship, while the recurring combination of positive serology and negative blood or CSF PCR supports models in which long-standing, immune−contained infection exerts subtle, compartmentalized effects through brain cysts, immune signaling, or past developmental insults. Integrating molecular markers with serological, clinical, and neurobiological data may help identify subgroups in whom infection contributes meaningfully to psychosis or mood symptoms.

### Biological mechanisms

The biological plausibility of a link between T. gondii infection and psychosis rests on the parasite’s neurotropic properties and its capacity to alter host neurochemistry and immune signaling. In keeping with the narrative scope of this mini-review, mechanistic inferences are drawn from two partially overlapping evidence streams: experimental animal and *in vitro* studies that allow direct manipulation of infection and neural pathways, and observational or biomarker studies in humans that provide correlational but clinically relevant data on inflammation, neurotransmission, and brain structure or function. Throughout this section, findings from rodent and other animal models (for example, alterations in fear conditioning, predator aversion, and dopaminergic signaling) are used primarily to illustrate plausible causal pathways, whereas human serological, genetic, immunological, and neuroimaging data are treated as supporting, higher-level evidence that these mechanisms may operate—albeit less directly—within clinical populations.

One frequently cited pathway involves dopaminergic dysregulation, as T. gondii encodes two genes analogous to host tyrosine hydroxylase, the rate−limiting enzyme in dopamine synthesis (29), and experimental work *in vitro* and in murine models indicates increased dopamine production within infected neural tissue, with potential consequences for reward processing, salience attribution, and psychotic experiences (30). A second major mechanism centers on neuroinflammation: chronic infection elicits sustained production of proinflammatory cytokines such as interleukin−6, tumor necrosis factor−α, and interferon−γ, which are implicated in microglial activation, synaptic remodeling, and the inflammatory profiles observed in schizophrenia and bipolar disorder (31). Neuroimaging and neuropathological data indicate preferential localization of cysts in limbic and prefrontal regions, including the amygdala, nucleus accumbens, and orbitofrontal cortex, areas central to emotion regulation, threat processing, and social cognition (32), and penetration of the blood–brain barrier by tachyzoites initiates immune cascades that facilitate cyst establishment followed by long-term, largely quiescent persistence with episodic reactivation under stress or immunosuppression (33). Host genetic variability further moderates risk, as polymorphisms in dopamine-related genes (for example, COMT Val158Met) and immune regulators (for example, IL−10, TNF−α) may interact with infection in line with diathesis–stress models where T. gondii functions as an environmental trigger on a vulnerable substrate (34). Convergent evidence from animal models shows that infected rodents exhibit reduced aversion to predator odor, increased exploration, and impairments in learning and fear conditioning, accompanied by dopamine elevation in ventral striatal regions (35), together outlining a mechanistic framework in which T. gondii influences neural systems central to psychosis through dopaminergic modulation, inflammatory signaling, and gene–environment interaction.

### Depression, anxiety, and autism spectrum co-morbidity

Beyond psychotic-spectrum disorders, accumulating research implicates Toxoplasma gondii infection in the pathophysiology of mood and anxiety disorders. Several epidemiological studies report higher T. gondii seropositivity in individuals with major depressive disorder, generalized anxiety disorder, and panic disorder compared with controls, and some data indicate greater anxiety sensitivity and reduced well-being in seropositive participants independent of psychotic symptoms (36–40). These associations are conceptually linked by shared inflammatory signatures across psychiatric illnesses: chronic T. gondii infection induces persistent, low-grade immune activation with elevated interleukin−6 and interferon−γ, cytokines also consistently increased in major depression and anxiety, and this proinflammatory milieu drives indoleamine−2,3−dioxygenase activation, diverting tryptophan toward kynurenine metabolism, reducing central serotonin availability and potentially contributing to anhedonia, fatigue, and mood dysregulation (41, 42).

An additional line of evidence implicates adaptive immune responses to neural antigens, particularly N−methyl−D−aspartate receptors (NMDARs), in the neuropsychiatric sequelae of toxoplasmosis. Experimental mouse work shows that chronic T. gondii infection can induce NMDAR autoantibodies accompanied by behavioral alterations, hippocampal pathology, and synaptic changes reminiscent of NMDAR encephalitis, suggesting that parasite-driven loss of immune tolerance to glutamatergic targets may be another route linking infection to disturbances in cognition, mood, and psychosis-like phenotypes (43). Clinical overlap between psychotic and depressive disorders further supports a transdiagnostic role, as psychotic depression shares dopaminergic and cytokine abnormalities with schizophrenia, and some longitudinal studies propose that T. gondii infection heightens susceptibility to both affective and psychotic symptoms depending on host genetics and immune response intensity (44). Although causality remains uncertain, converging immunological, neurochemical, and behavioral findings position T. gondii as a potential unifying biological factor linking mood and psychotic pathologies within an infection–immunity–behavior framework.

Several authors have also explored possible links between T. gondii infection and autism spectrum disorders (ASD), though current evidence remains preliminary and non-causal. In a comprehensive review, Prandota (45) suggests that congenital and/or chronic cerebral toxoplasmosis might help explain some of the metabolic, immune, epigenetic, endocrine, and phenotypic abnormalities described in ASD, Down syndrome, and Alzheimer’s disease, proposing that impaired sulfation and sulfoxidation, altered DHEA–DHEA-S balance, disturbed tryptophan and carbohydrate metabolism, chronic vascular neuroinflammation, and hormone dysregulation in ASD could, in part, reflect host responses to persistent cerebral infection, with parasite–host interactions at the level of sulfated proteoglycans, phosphorylation, chromatin structure, and DNA methylation offering putative mechanistic links to atypical neurodevelopment. Abdoli and Dalimi (46) examine the toxoplasmosis–autism relationship through the “extreme male brain” framework, proposing that latent T. gondii infection may be associated with elevated testosterone and could therefore contribute to ASD risk in genetically susceptible offspring; drawing on reports of higher testosterone in seropositive men and in experimentally infected animals, they hypothesize that analogous endocrine effects in pregnant women might increase fetal androgen exposure during critical neurodevelopmental windows and align with findings of elevated prenatal Δ4 sex steroids in ASD, while explicitly framing this as a speculative, hypothesis-generating model rather than established evidence of causality.

In another study, Elzeky et al. (47) examined toxoplasmosis in Egyptian children with a spectrum of neurodevelopmental disorders using serology, PCR, and genotyping. Children with conditions such as hydrocephalus, epilepsy, cerebral palsy, and intellectual disability showed markedly higher T. gondii seroprevalence, elevated IgG titers, and, in a subset, detectable parasite DNA in blood compared with neurologically healthy controls, indicating that both past and possibly ongoing infection are overrepresented in this clinical population. Genotyping of GRA6-positive samples revealed a predominance of virulent type I strains alongside atypical genotypes, leading the authors to suggest that, in this setting, strain biology interacting with host and environmental factors may help explain clustering of toxoplasmosis with severe neurodevelopmental outcomes such as hydrocephalus and epilepsy, even though clear strain–phenotype specificity could not be established.

### Methodological challenges and limitations

Interpretation of associations between Toxoplasma gondii, cat ownership, and mental health outcomes is constrained by several methodological issues that limit strong causal inference. Serological testing is intrinsically ambiguous: IgG indicates prior exposure without confirming current infection or cerebral cyst burden, whereas IgM suggests recent infection yet is prone to false positives due to cross−reactivity or slow decline, so single time−point antibody measures can misclassify infection timing and obscure whether exposure preceded symptom onset. Longitudinal serological designs remain relatively uncommon, further restricting insight into temporal dynamics of infection and symptom emergence. Retrospective self−report of cat ownership and contact frequency introduces recall bias, and individuals with psychotic or affective symptoms may differentially reconstruct childhood pet histories, potentially exaggerating observed associations (19). Confounding by indication represents an additional concern: people with emerging social withdrawal or loneliness—a frequent prodrome of psychosis and depression—may be more inclined to acquire cats for emotional support, making cat exposure as much a consequence as a putative cause of psychopathology. Publication bias likely distorts the literature, as studies reporting positive associations between infection and psychiatric outcomes are more likely to be published than null findings, inflating effect sizes in meta−analyses (48, 49). Cross−national differences in cat−keeping practices, dietary customs, and environmental conditions also generate heterogeneous exposure risks; for example, settings with predominantly indoor cats and routine veterinary care may have far lower T. gondii transmission than regions with large outdoor or feral cat populations (50). These cultural and ecological variations underscore the need for contextual sensitivity when comparing international data and mean that, without addressing such limitations, interpretations of the T. gondii–psychosis link must remain cautious and tentative.

### Alternative explanations and criticisms

Despite accumulating evidence for associations between T. gondii exposure and psychotic symptoms, several alternative explanations and critical perspectives must be considered. Reverse causation is a central concern: individuals exhibiting early or subclinical psychotic features, social withdrawal, or affective distress may be particularly drawn to companion animals, including cats, as sources of comfort and social connection, creating a spurious pattern in which psychiatric vulnerability predicts cat ownership rather than results from it. Depressive and anxious traits can similarly foster stronger attachment to pets, further blurring directionality in observed relationships. Third variables also complicate causal interpretations; urban living, for example, is a well−established environmental risk factor for schizophrenia (51) and simultaneously increases exposure opportunities to domestic cats, urban wildlife reservoirs of T. gondii, contaminated soil, and variable sanitation conditions. In rural environments, agricultural activities and frequent soil contact may play a larger role in transmission than household cats (52), while dietary habits, food safety practices, and local climate further shape infection prevalence independently of pet ownership. These overlapping ecological and social factors highlight the importance of multifactorial models that integrate infectious, environmental, and psychosocial determinants of psychosis risk rather than privileging a single exposure. Finally, discourse around cat ownership and mental illness interacts with entrenched cultural stereotypes—most notably the “cat lady” trope (53)—which risk reinforcing stigma toward both psychiatric populations and pet owners, making it essential that scientific communication avoids deterministic or sensational narratives and clearly differentiates modest, probabilistic risk signals from pathologizing ordinary human–animal companionship.

## Conclusion

The evidence suggests that Toxoplasma gondii modestly increases risk for psychotic-spectrum symptoms, with more variable but biologically plausible links to depression and anxiety via inflammatory and tryptophan–kynurenine pathways. Cat ownership functions as an imperfect, context-dependent proxy for exposure, with associations often attenuating after adjustment, indicating cats are neither necessary nor sufficient as a causal factor. It is also important to distinguish that much of the fine-grained mechanistic detail (e.g., parasite-driven dopamine synthesis and behavioral manipulation in response to predator cues) comes from animal and *in vitro* work, whereas human studies mainly document broader patterns of elevated inflammatory markers and altered tryptophan–kynurenine metabolism that are compatible with, but do not definitively prove, these causal pathways. Clinically, emphasis should remain on basic preventive hygiene and targeted, mechanism-informed research rather than routine screening or alarmist messaging.
